# The Spectral X-ray Imaging Data Acquisition (SpeXIDAQ) Framework

**DOI:** 10.3390/s21020563

**Published:** 2021-01-14

**Authors:** Frederic Van Assche, Sander Vanheule, Luc Van Hoorebeke, Matthieu N. Boone

**Affiliations:** Radiation Physics Research Group—UGCT, Department of Physics and Astronomy, Ghent University, Proeftuinstraat 86/N12, B-9000 Ghent, Belgium; sander.vanheule@ugent.be (S.V.); luc.vanhoorebeke@ugent.be (L.V.H.); matthieu.boone@ugent.be (M.N.B.)

**Keywords:** hyperspectral X-ray imaging, photon counting detectors, image processing, visualization

## Abstract

Photon counting X-ray imagers have found their way into the mainstream scientific community in recent years, and have become important components in many scientific setups. These camera systems are in active development, with output data rates increasing significantly with every new generation of devices. A different class of PCD (Photon Counting Detector) devices has become generally available, where camera data output is no longer a matrix of photon counts but instead direct measurements of the deposited charge per pixel in every frame, which requires significant off-camera processing. This type of PCD, called a hyperspectral X-ray camera due to its fully spectroscopic output, yet again increases the demands put on the acquisition and processing backend. Not only are bandwidth requirements increased, but the need to do extensive data processing is also introduced with these hyperspectral PCD devices. To cope with these new developments the Spectral X-ray Imaging Data Acquisition framework (SpeXIDAQ) has been developed. All aspects of the imaging pipeline are handled by the SpeXIDAQ framework: from detector control and frame grabbing, to processing, storage and live visualisation during experiments.

## 1. Introduction

In recent years a new variant of photon counting X-ray sensors, the hyperspectral or fully energy resolving sensors, have become sufficiently mature to find their way into actual scientific and industrial applications. The detailed energy information provided by these systems allows for improved measurements in existing applications, as well as the emergence of completely novel application domains. The main advantage of the spectral content in the images is the possibility of material identification at lab X-ray sources. Due to their construction as full-field pixellated devices, these cameras allow for capture of chemical information not only in X-ray fluorescence (XRF) applications, but also in full-field transmission imaging [1,2] and transmission computed tomography (CT) [3,4,5]. Even in the established application domain of XRF imaging, these hyperspectral systems provide added value through their 2D nature as opposed to the single-element energy dispersive detectors commonly used in XRF measurement setups, allowing for improvements in speed and image quality for fluorescence measurements as well [6,7,8]. The biggest potential of hyperspectral X-ray sensors is to be found in their ability to bring energy information to the lab, without the non-trivial requirement of tuneable monochromatic X-ray sources. However, even at synchrotron and X-ray free-electron lasers (XFEL) [9] facilities hyperspectral cameras can be combined with established imaging techniques to enable novel experiments to take place, such as in hyperspectral dark-field X-ray imaging [10], or hyperspectral ptychographic imaging [11]. Finally, these hyperspectral systems also provide new diagnostic tools for lab setups and beamlines, offering accurate measurement of source spectra and optics parameters, as demonstrated previously by the authors [12].

The emergence of hyperspectral X-ray sensors has required a departure from the conventional approach to operating X-ray imaging systems. These sensors no longer only count the incoming photons on the device based on per pixel detection thresholds, but output the actual measured charge clusters in each frame and pixel, which can be reconstructed to accurate descriptions of single photon events. This has placed a much greater emphasis on off-camera data processing as would be the case with more conventional sensors. Both the bandwidth required and the complexity of the processing is greatly increased in order to support the fast framerates of such systems (hundreds to thousands of Hz for current generation devices), and to reconstruct the incident photon information from the collected charges. In next generation devices, which are expected to increase framerates and bandwidth requirements up to two orders of magnitude, these requirements will only become more pressing if one wants to keep the ability for continuous, real-time operation instead of burst mode acquisition.

The currently available hyperspectral X-ray cameras have no to limited software support, with only simplified functionality and are in many cases difficult or impossible to integrate into bigger measurement setups. To counter the shortcomings of the current state, and to anticipate future developments in sensor capabilities, a new software framework was developed at Ghent University: the Spectral X-ray Imaging Data Acquisition framework, or SpeXIDAQ.

## 2. Architectural Overview

### 2.1. General Structure

The SpeXIDAQ system is implemented as a set of separate, network connected binaries. Each binary performs a specific task in the acquisition and processing chain, and all parts are connected using the ZeroMQ (https://zeromq.org) message passing framework. The main advantage of this implementation style is the full scalability offered by the decoupling of processing steps. The entire framework can run on a single modest workstation for lower data rate detectors, or can be distributed over multiple machines in a small cluster for high performance applications. This scaling of available performance can even be performed on a live and fully functional system, by spinning up or shutting down worker processes. Any reconfiguration of the acquisition chain is handled transparently by the network layer, with available data being distributed on-the-fly according to the processing power of the individual nodes in the network.

A second advantage of the network-transparent nature is the immediate availability of full remote control from multiple concurrent clients. Nearly all aspects of a running installation can be controlled using graphical user interfaces (GUIs) that connect over the network, with support for multiple active clients. This way the data acquisition (DAQ) system can be integrated in an existing experimental setup as a fully headless and independent system, with just the user-facing GUI applications installed on the workstations used for experiment control and monitoring. This general structure of the SpeXIDAQ framework is not unlike how EPICS and GDA [13] are being used at Diamond Light Source, albeit much more restricted and domain-specific in scope. This pattern of a high performance backend handling hardware and data processing, coupled with a science-oriented GUI toolkit is a very successful model for complex scientific devices.

### 2.2. The Default Processing Chain

**Figure 1:**
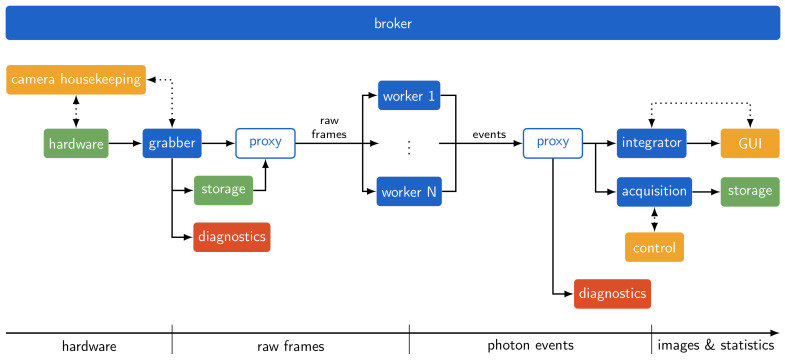
Schematic representation of an example configuration using the new data flow system. The blocks represent isolated processes or hardware elements, all communicating using ZeroMQ message streams. Solid lines represent image and sensor data, dotted lines indicate control and metadata streams. Blue elements are headless SpeXIDAQ processes, green elements indicate hardware parts, red elements are optional diagnostic and debugging tools, and orange blocks represent user-facing utilities with graphical interfaces.

The basic layout of a functioning SpeXIDAQ configuration is outlined in Figure 1. The different software elements and their functions are described in Section 3. This default layout combined with one of the supported camera systems handles hardware control, frame conditioning and processing, integration of camera frames into final output images, and flexible measurements with data storage on disk. For most experiments this default implementation is sufficient and provides a wealth of experimental information for analysis and experiment-specific offline processing.

### 2.3. Customisability

Because of the wealth of new information provided by hyperspectral X-ray cameras compared to their more conventional counterparts, the default processing workflow is not always desired. Depending on the specific imaging technique used for a scientific experiment, different ways of processing the camera output can provide tangible advantages in camera performance, and highly specialized ways of integrating the SpeXIDAQ system with other experiment setups can be required.

In case the default workflow is not well suited to an experiment, or more advanced functionality is desired, significant possibilities for data processing customization and external communication are provided. This customization support is provided by well-constrained C++ APIs, which allow writing small plugins that can be loaded into the various components of the SpeXIDAQ system. The different available plugin types are listed as part of the implementation description in Section 3, along with the SpeXIDAQ process they are loaded into. Some of the plugin types are optional and only required for more advanced use cases, such as the exposure and eventfilter plugins. Of the mandatory plugins, most are supplied with a default implementation in a standard SpeXIDAQ installation, with the grabber plugin type being the exception. This plugin is completely hardware-dependent, and has to be developed for each hyperspectral camera or camera family that should be supported by SpeXIDAQ. Depending on the specifics of certain cameras, some of the other plugin types can benefit from a more specialized implementation.

## 3. Implementation

The SpeXIDAQ framework consists of a number of mandatory and optional modules. The general structure of the system is that of a set of individual processes which each perform a specific and well-defined task. The hardware support and customisation is implemented in the form of external plugins, dynamically loaded on-demand as required by the active configuration.

### 3.1. The Broker Process

The broker process is required by all other elements. This process contains the active configuration and calibration data, and keeps track of the existence of all other elements in the processing chain. Any process providing a data stream registers this stream with the broker. All other processes and GUI clients connect to the broker to receive the configuration, calibration and network topology.

### 3.2. The Frame Grabber Process

Camera control and frame extraction is handled by the grabber process. Nearly all functionality is part of the mandatory hardware support plugin, with the grabber process itself only providing internal frame queue management and the API required by the hardware support plugins.

*Grabber plugin (mandatory):*

A plugin of the grabber type implements the required hardware control and data transfer functionality for a specific hyperspectral camera or family of cameras. These plugins can be limited to just a data receiver role with control and monitoring handled by firmware or separate software. The source of incoming frames is not required to be a physical detector. For example, a simple plugin reading raw frames from disk is available which can be used to replay previous stored measurements. This is useful in case one is developing new plugins further downstream and a reproducible input is desired, or a stored experiment has to be re-analyzed or viewed again through the GUI.

### 3.3. Worker Processes

One or more worker processes subscribe to raw detector data as input, and process these raw frames into a stream of photon events. As part of this processing, the detector calibration is applied to the incoming frames.

*Processing plugin (mandatory):*

A processing plugin performs the necessary frame conditioning and cluster finding on the incoming raw frames. Most hardware can take advantage of the default implementation included with SpeXIDAQ, which includes dark current removal, noise thresholding and a basic, efficient implementation of non-biased cluster finding in the form of a recursive flood-fill algorithm.

### 3.4. The Proxy Process

The proxy process handles the distribution of the workload over the different worker instances. It has two main responsibilities. In the first it takes raw frames sent out by the grabber process and distributes it over the available worker processes according to their individual throughput. In the second it combines the multiple photon event streams being output by the worker processes into a single stream for other modules further downstream.

### 3.5. The Live and Storage Integration Processes

Two different integrating processes are running in a default SpeXIDAQ configuration. Both processes take the stream of photon events emitted by the worker processes, and turn these events into scientifically useful output such as images and histograms.

The integrator process integrates the event stream into live preview images and data for all GUI clients monitoring and controlling the experiment. The parameters of the different integration and data types can be modified from all GUI instances on the network.

This functionality is duplicated in the acquisition process, which also integrates the incoming photon events into the same output types as the integrator process. Instead of using these integrations for live feedback in the GUI, the results are saved to storage. It is this process that performs the actual quantitative measurements.

By splitting the integration over two processes respectively handling live preview and stored measurements, the live monitoring in the user interface is completely decoupled from the actual measurements. While a measurement is active in the acquisition process, a user can still independently change the parameters of the integrator process for monitoring and previewing the ongoing experiment.

*Integration plugin (multiple, mandatory):*

A multitude of integration plugins is provided with the default installation. These plugins perform the actual integration of the stream of photon events into various images and histograms, both for the live previews in the GUI as well as the data stored to disk during an actual acquisition. Each data type described in Section 4.1 is implemented as one such integration plugin.

*Eventfilter plugin (optional):*

The optional eventfilter plugin type has the ability to modify the stream of photon events before further processing. This plugin type receives all reconstructed photon events grouped by frame in the internal SpeXIDAQ data format, and can correct or remove individual events as required. This plugin type is also able to modify the metadata of the processed frames, enabling advanced functionality such as a dynamic region-of-interest, interpolation, etc. An advanced operating mode of hyperspectral X-ray sensors with small (sub-50 μm) pixels uses the precise measurement of deposited charge clusters over multiple pixels to reconstruct the incident photon position on a grid with a pitch smaller than the physical pixel grid. This technique is called sub-pixel resolution [14,15], and is implemented as camera-specific eventfilter plugins in the SpeXIDAQ framework. Using such a sub-pixel plugin can provide higher spatial resolution for both live viewing and stored measurements.

*Exposure plugin (optional):*

The optional exposure plugin type is used to compensate for non-uniform or unpredictable exposures. The function of this plugin type is to provide a per-pixel counter of effectively exposed frames. This effective exposure map is used to normalize the exposure when integrating individual frames into final output images. Usage examples include pulsed sources such as free electron lasers, and any kind of non-uniform time varying exposure.

### 3.6. The External Control Process

The control process facilitates automated external control of the quantitative acquisitions, using hardware solutions such as transistor-transistor logic (TTL) trigger inputs, or network-based external control. This offers the possibility of tight integration into existing measurement setups. This process as a whole is optional in case no external automation is desired, and stand-alone operation using the GUI is sufficient for the application.

*Control plugin (mandatory, if external control used):*

In case external control is desired, a control plugin is required to implement the actual external interface. Such a plugin could for example integrate external hardware inputs to synchronise SpeXIDAQ measurements with a larger setup, or provide a network socket through which third-party software can automate and monitor the data acquisition.

## 4. Data Output Types and Storage Formats

### 4.1. Output Types

By default the processing chain outputs a number of different views and datatypes, all of them available both for live viewing in the main GUI, and in controlled measurements which are sent to storage.

#### 4.1.1. Integrated Counts and Integrated Energy

The integrated counts datatype provides the behaviour most closely emulating a regular photon counting detector. It provides images where the pixel values represent the total number of photons detected during an integration. The closely related integrated energy view sums the deposited energy in keV for each reconstructed photon over an exposure in a floating point image. By recording dose rather than counts, this data type more closely resembles the output of an integrating detector, and is less susceptible to pulse pile-up effects. It should be noted that the integrated energy differs subtly from a classical energy integrating detector, which would integrate the deposited energy without reconstructing charge clusters and assigning the full deposited energy to their center of mass. By including the cluster reconstruction step this view will still be affected by very heavy pulse pile-up in case large numbers of photons are incorrectly reconstructed as a small number of very large charge clusters. However, this situation is unlikely to occur in normal operation.

Both views serve similar roles by providing a quick high-statistics view of what the camera sees, for example during sample positioning. In cases of high flux and thus high pulse pile-up, the integrated counts view can differ noticeably from the integrated energy view.

#### 4.1.2. Photon Spectrum

The photon spectrum output type is a histogram of photon counts per energy, usually presented as a line plot on a linear or logarithmic counts scale. Using this data type the incoming energy spectrum can be examined at a global level, for example when identifying materials or while configuring a source with tuneable energy spectrum.

**Figure 2:**
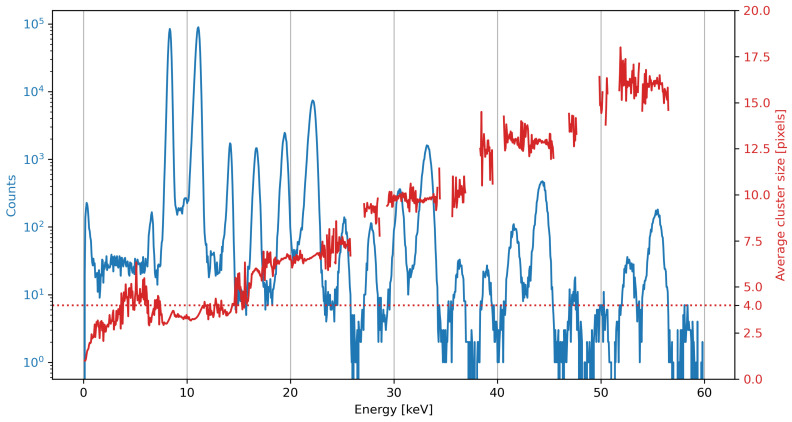
A recorded beamline spectrum showing both photon counts and average charge cluster sizes over a 60 keV interval. Beyond the peak at 14 keV no significant amount of photons are expected in the beam spectrum. The spectrum shows a pink beam spectrum at the Diamond Light Source I13-1 coherence branch recorded with the SLcam, with a global flux of $4.9\times 10^{3}$ $\mathrm{ph}s^{-1}$ ${\mathrm{cm}}^{-2}$ and a global sensor pixel occupancy around 0.1%. The appearance of peaks at pile-up energies is caused in part by small amounts of higher-order beam harmonics, and the fact that this illumination was not spatially homogeneous, with local brightness peaks of pixel occupancies of 3% and higher included in the measured spectrum. The separation of pile-up events from undulator harmonics is non-trivial.

An alternative output is also available which provides the average charge cluster size in relation to event energy. This is mostly a diagnostic tool to identify energy ranges with higher pulse pile-up, and by result larger charge clusters. By combining the photon spectrum output in counts and average cluster size views the incident energy spectrum can be analysed live or offline in great detail, identifying physical emission peaks, sensor artefacts, and pulse pile-up peaks, as illustrated in Figure 2. This figure shows the reconstructed beam spectrum in photon counts and average charge cluster size. While no physical photons are expected above 14 keV in this configuration of the beamline, a significant number of events is still reconstructed up to much higher energies. These are pulse pile-up peaks, as identified by the strong increase in average charge cluster size for each next integer multiple of the true photon energies. The pnCCD’s normal single photon charge clusters are roughly 4 pixels in size [16], indicated by the dotted line on Figure 2.

#### 4.1.3. Mean Energy

**Figure 3:**
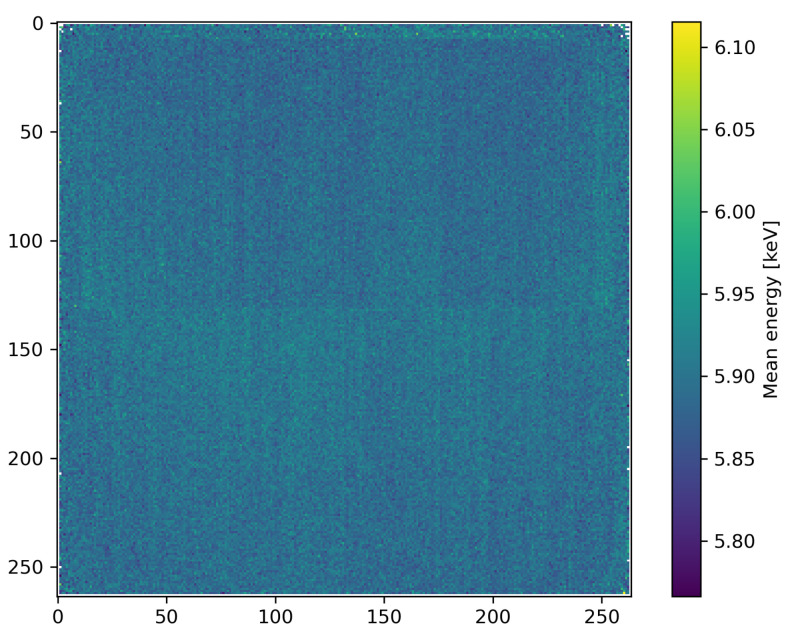
Example of the mean energy view, showing the mean photon energy of all events in the range 5.75 to 6.15 keV, which includes the 5.9 keV Mn $K_{\alpha }$ peak of a ${\mathrm{Fe}}^{55}$ calibration source. The mean energy view shows very small variations in spectrum, highlighting the structure of the pnCCD sensor in a slight mismatch of calibration. The Fe^55^ calibration source used for this figure is very weak with an overall flux below $1\times 10^{3}$ $\mathrm{ph}s^{-1}$ ${\mathrm{cm}}^{-2}$, leading to negligible pile-up.

The mean energy view provides a pixel mapping of the mean energy of all photons within a configurable energy range. This is a strong diagnostic tool that can visualize small spatial differences in the incident spectrum. These small variations can be used as a sensor performance and calibration quality verification tool as shown in Figure 3, but can also carry valuable measurement information, for example visualizing the effect of beam hardening through a sample, or spatial variations in the spectral properties of the X-ray beam.

#### 4.1.4. False Colour

**Figure 4:**
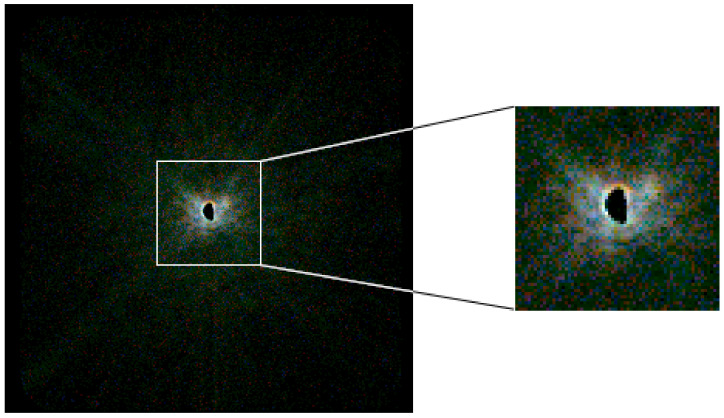
Example of the false colour output being used during a ptychography beamtime. The pink spectrum of the beam has been split over the red, green and blue ranges and visualizes the energy-dependent diffraction caused by the optics used during the beamtime. The three colour planes have been autoscaled to fill the full 8 bit dynamic range of each colour on a logarithmic scale. The red, green and blue energy ranges are respectively 6.8 to 7.6 keV, 7.6 to 8.4 keV, and 8.4 to 9.2 keV. A two minute exposure time was used, and the global flux was $3.1\times 10^{3}$ $\mathrm{ph}s^{-1}$ ${\mathrm{cm}}^{-2}$, with hotspots of $9.6\times 10^{4}$ $\mathrm{ph}s^{-1}$ ${\mathrm{cm}}^{-2}$.

While it is very challenging to visualize the inherently three dimensional (sensor X, sensor Y, energy) dataset as one image on a two dimensional display, the false colour view provides a compromise between the difficult to present full spectral data and the conventional grayscale views of non-hyperspectral X-ray cameras. The experimenter is able to configure up to three energy ranges which are then represented as the red, green, and blue channels of a colour 2D image. This provides the experimenter with a view that is carrying true spectral information, while still remaining straightforward to interpret visually, such as the example shown in Figure 4.

#### 4.1.5. Data Cube

The full advantage of hyperspectral X-ray cameras is realised using the data cube output type, which generates a series of 2D images at configurable energy intervals. Typically these intervals are about an order of magnitude narrower than the energy resolution of the sensor, and the first and last energy bin is chosen according to the spectral region of interest. In case of a white X-ray beam it is not uncommon to have multiple thousands of narrow energy bins, which can of course always be rebinned for easier offline analysis and reduced storage space.

#### 4.1.6. Raw Detector Output

The raw detector output can be stored to disk in an efficient binary format, given sufficient storage bandwidth is available. This allows for offline reprocessing of the acquired data after performing a measurement, and is an invaluable tool in the case of hard to repeat experiments, for example at synchrotron beamlines. Because the detector output is stored fully unprocessed, there is even the possibility of taking advantage of improvements in calibration and processing algorithms at a later date, and any features not currently implemented by the SpeXIDAQ framework or custom plugins developed by the experimenter can still be applied to the stored raw data as they become available.

It should be noted that the storage bandwidth demands of hyperspectral X-ray sensors can be significant, exceeding the performance offered by single hard disk drives. For example, a four-sensor HEXITEC configuration clocked at 6.3 kHz requires around 2.5 $\mathrm{Gbit}s^{-1}$ of bandwidth, which is challenging for common configurations of hard disk drives, even in RAID configurations, at time of writing. With future ASICs approaching 100 $\mathrm{Gbit}s^{-1}$ rates, storage of raw output for archival purposes will unfortunately become impossible for most experimental setups. At the same time the quantity of data generated even for short experiments is not well suited to the smaller capacities of solid state drives at time of writing, even though they could offer the required bandwidth. Nevertheless the option is available, and even without proper bandwidth a subset of the recorded frames is still saved including a frame counter to indicate missed frames. This allows for diagnostic and development use of raw detector output even for very high bandwidth cameras in short bursts.

### 4.2. Storage Formats

SpeXIDAQ offers the option to select either TIFF or HDF5 output for the controlled measurements, and this for every individual measurement performed during an experiment. Regardless of the chosen storage format, a few human readable files are stored with every measurement which contain the relevant configuration and metadata.

#### 4.2.1. TIFF Output

The TIFF output option generates a TIFF file for every 2D image output type enabled during a measurement. For example, the data cube output type will generate one TIFF image for every energy bin configured. The data types which output a 1D dataset, such as the photon spectrum output, are stored in tab-separated text files.

#### 4.2.2. HDF5 Output

When HDF5 output is selected, each measurement is saved in its own HDF5 file. This file contains all enabled output types according to their dimensionality. The data cube output is stored as a true 3D dataset, images as 2D arrays, and the photon spectrum and related output as 1D arrays. The HDF5 file also contains the metadata and configuration as properties in order to be fully self-contained. In cases where one dataset axis represents energy bins, a separate dataset is also stored which contains the lower bound energy of each energy bin.

#### 4.2.3. Optional Raw Frame Storage Format

In addition and parallel to the above described processed data outputs, it is also possible to store the camera’s unprocessed output, if storage bandwidth exceeds the requirements set by the used hyperspectral camera. When raw camera output storage is enabled, these frames are stored in a .spexiraw file. This is a simple binary format containing a small header with some metadata, followed by the detector output frames in unprocessed and uncompressed form.

## 5. Main Features

### 5.1. Supported Hardware

The SpeXIDAQ system supports, at time of publication, the pnCCD based SLcam system and the HEXITEC family of hyperspectral X-ray cameras. Through their respective strengths and applications these two device families provide good coverage of the currently available hyperspectral X-ray imaging landscape.

#### 5.1.1. The pnCCD (SLcam)

The SLcam system [17] is based on a 264×264 pixel, 450 μm thick fully depleted silicon CCD sensor with 48 μm pixel pitch, clocked at 400 Hz ( 1 kHz optional upgrade). Thanks to its exceptionally low readout noise an energy resolution of 144 eV full-width half-maximum (FWHM) at 6 keV can be achieved in general purpose operation using the SpeXIDAQ calibration techniques and processing algorithms.

Its very strong spectral performance does come at a price: maximum flux rates are on the order of $5\times 10^{5}$ $s^{-1}$ ${\mathrm{cm}}^{-2}$ before pulse pile-up becomes too significant, and the use of a silicon active medium limits the useful energy range to not much more than 30 keV.

Because of the higher complexity of the camera housekeeping functionality, this part of the pnCCD support is handled by a separate housekeeping application. The SpeXIDAQ plugin is very limited in scope, as it just reads out frames from the ADCs and pushes them into the SpeXIDAQ pipeline.

#### 5.1.2. The HEXITEC Family

The HEXITEC hyperspectral X-ray ASIC [18] offers 80×80 pixels at 250 μm pitch, read out at up to 9 kHz. The ASIC is often coupled to a 2 mm CdTe sensor, but can also be provided with GaAs and CZT [19] active media. In its 2 mm CdTe configuration the HEXITEC ASICs achieve an energy resolution of around 1 keV FWHM at 60 keV using SpeXIDAQ, and the full design 9 kHz readout speed is available real-time on a modest dedicated workstation.

Through use of high-Z sensor materials, the HEXITEC family offers a working energy range up to 200 keV, and their higher readoud speeds significantly increase acceptable flux rates compared to the pnCCD system. The relatively limited pixel count (80×80) is a limiting factor for imaging, which can be solved by using a tiled sensor configuration thanks to the three side buttability of the HEXITEC ASIC.

A default SpeXIDAQ installation supports the commercially available single ASIC HEXITEC system out of the box, as well as the currently in development four sensor array (in 2×2 configuration) system [20]. The SpeXIDAQ plugin for HEXITEC support is entirely self-contained, handling everything from hardware control to frame readout. Future variants in the HEXITEC family, such as a 24 sensor system [21] can also benefit from the existing HEXITEC support in SpeXIDAQ.

### 5.2. Software Features

#### 5.2.1. Backend Features

A large part of the flexibility of the SpeXIDAQ backend is designed into the broker process, which contains the active configuration and calibration. By switching configurations, an operator can change from one camera to another, or choose between different configurations of a single camera for experiment specific optimizations. Any change in configuration—both by choosing a different one, or by changing the configuration parameters in the INI files on disk—are immediately propagated to all other processes and GUI clients.

In turn all other processes and GUI clients require only the address of a broker to connect to, and will take their place in the processing pipeline automatically. For example, changing the number of worker processes used to read raw frames and output photon events can be changed on-the-fly by stopping or starting instances. The processing load will be split automatically over the available processes in function of their respective processing power, with worker instances on slower machines receiving proportionally fewer frames to process.

This is the essence of the distributed and modular architecture behind the SpeXIDAQ system, and highlights how it can scale to next-generation hyperspectral X-ray cameras where a single workstation will no longer suffice for data processing.

Also included with the backend are a series of command line (CLI) tools which allow an operator or camera developer to extract a wealth of intermediate and final data for diagnostic purposes. At various points in the processing workflow the results can be observed, which is invaluable in the case one is developing a SpeXIDAQ plugin, or working on new hardware support. These diagnostic utilities are also at time of publishing central to the calibration process of a new device.

#### 5.2.2. System Integration Features

Through the control plugins described in Section 3, measurements can be controlled by an external source. This external control can be as simple as a hardware TTL trigger signalling the start of the next measurement, or can involve the external system pushing an entire measurement configuration over a network connection. Both of these extremes have been used successfully over many experiments and tens of thousands of individual measurements.

The flexibility of these external control implementations allows for rapid integration on one hand, for example using TTL triggers during a beamtime, and deep integration into existing control systems in case of a fixed setup on the other hand. Stand-alone manual operation remains possible at all times, even when a control plugin is being used.

This integration can be two-way, even when using hardware TTL triggers. The SpeXIDAQ framework is for example able to assert a TTL output high whenever it is waiting for the next start trigger. By using this “ready” signal a two-way synchronised measurement system can be constructed together with an external control system. This external control system waits for SpeXIDAQ to be ready and waiting, performs its next tasks such as altering the sample position, then sends a start trigger to SpeXIDAQ. Once the measurement is finished the ready signal becomes active again, after which the cycle can repeat until the experiment is finished.

#### 5.2.3. Measurement Features

Measurements are assigned a name under which they are stored, either auto-generated based on the current date and time, or specified by the operator or external control. Each measurement contains one or more of the data types described in Section 4.1, and the specific parameters of each of these data types are configured in the GUI and stored in the measurement metadata. A number of global measurement settings are provided as well, including an integration time, and minimal and maximal charge cluster sizes. Through a comment input field the operator can specify experiment parameters, remarks, sample information, and any other relevant details. All of the provided parameters are stored together with the output data for future reference.

The charge cluster range selection feature allows the operator to optimize the included photon events, for example favouring linearity over spectral correctness by not imposing any limits. By placing more restrictive limits on accepted photon events, the energy response can be improved at the expense of linearity by artificially lowering recorded counts in cases of high pile-up, but removing or strongly reducing pile-up spectral peaks aiding material identification efforts. This selection is usually done based on the requirements of the specific application and measurement goal.

To enable better integration into existing measurement setups and techniques, measurements can contain multiple sub-measurements or steps. By default a measurement is just a single integration, but when performing for example a tomography experiment where *N* projections need to be acquired, a measurement can be started consisting of *N* steps, each being triggered to start after the sample has been rotated to the next projection angle.

#### 5.2.4. GUI Features

**Figure 5:**
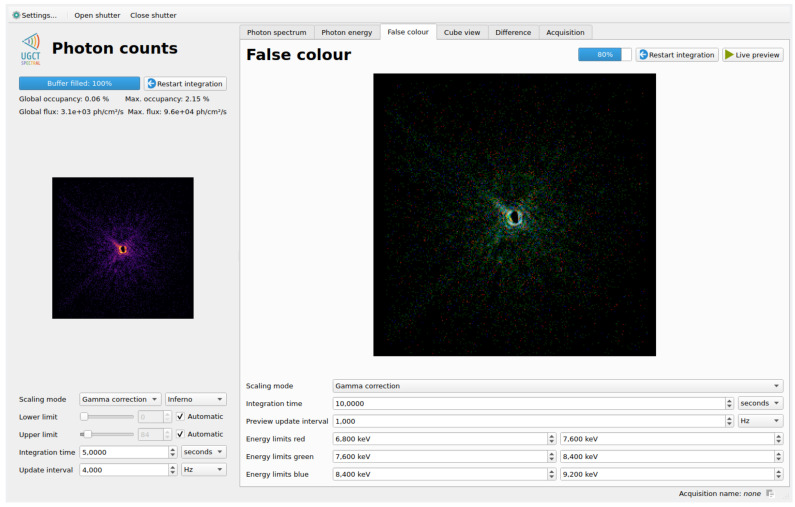
The main user interface used to control and monitor the SpeXIDAQ system. This example shows the pnCCD-based SLcam being used at a ptychography experiment at Diamond Light Source I13-1.

The main graphical interface for the SpeXIDAQ system serves both as a feature-rich tool to monitor the live camera output and scientific observables, and contains the controls used to configure and start recorded measurements. Figure 5 shows the main GUI being used during an X-ray ptychography experiment. The left hand side of the application is dedicated to the integrated counts view with various visualisation and integration time controls. The right hand side contains the other data output types outlined in Section 4.1, presented as a series of tabs. In Figure 5 the false colour or RGB view is selected, showing the spectral nature of the detector. Also included in the GUI is an indication of the incoming flux and the percentage of pixels over threshold in each frame (called occupancy), both across the entire sensor, and in the brightest hotspot. These numbers are commonly used to optimize the incoming flux to a level minimizing pulse pile-up and maximizing statistics for a given integration time.

**Figure 6:**
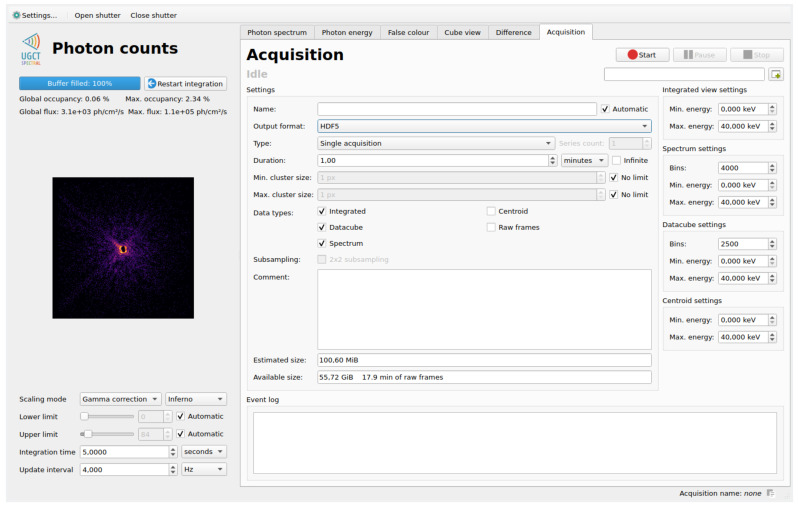
The measurement control section of the main user interface. Through this interface the operator configures and starts the controlled measurements which are sent to storage, and which run completely independently of all other viewing features of the GUI.

Also part of the main GUI is the measurement control interface, shown in Figure 6. These controls allow the camera operator to select the desired output data types with their respective parameters, the total integration time, and various other camera settings. An estimate of the required storage space is provided, along with a free storage space indicator.

While a measurement is active, all live preview functionality of the GUI is still available. In other words, any active measurement is completely independent from the settings and actions available for live viewing, allowing for continuous investigation and monitoring during long experiments.

## 6. Performance

All of the data processing was performed on a workstation assembled using recent off-the-shelf hardware with a total budget around $2500, of which roughly half was used for storage media. The main parameters of the system are outlined in Table 1. This configuration provides more than enough headroom to run a HEXITEC or SLcam system at full rate, including an active measurement and at least one GUI instance set to high refresh rates for live viewing.

**Figure 7:**
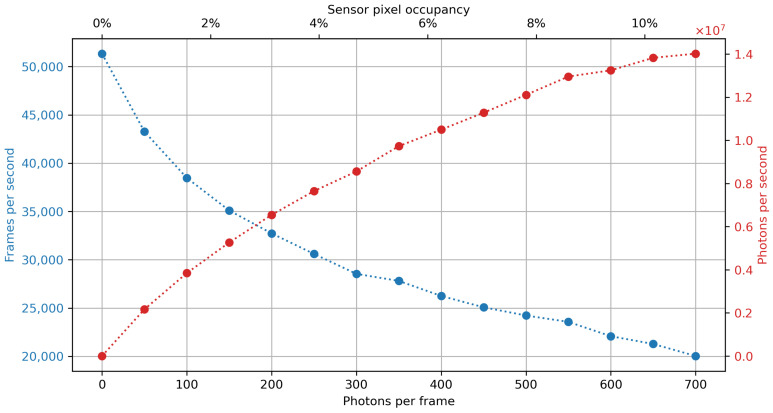
Maximum throughput in frames per second for HEXITEC-type frames at varying photon counts per frame. This benchmark shows the single-core performance of the SpeXIDAQ implementation, and can scale roughly by the amount of CPU cores. The flood-fill algorithm is moderately sensitive to the total count of reconstructed events per frame, with the decrease in throughput flattening out as pile-up becomes the dominant source of reconstructed events.

The currently implemented flood-fill cluster finding algorithm has a running time that depends on the overall number of photons per frame. This effect is demonstrated in Figure 7. The figure shows the maximum throughput in frames per second for real-world HEXITEC frames (80×80 pixels) at various pixel occupancy levels. All testing was performed on the workstation outlined in Table 1. The entire pipeline also gets slightly more efficient in terms of photons processed per second as frame sizes increase because of a fixed additional overhead associated with the frame-based data transfers between processes, which are independent of pixel count and flux.

Finally, the resources used by the entire software stack are outlined in Table 2, where an active SpeXIDAQ installation is monitored for the HEXITEC and SLcam detector. In each instance, an active measurement is being performed with 4000 energy bins in the data cube output type. Memory consumption can be lowered by decreasing the number of energy bins, which is possible for both the measurement backend and the live GUI independently. While the SLcam only requires half the bandwidth (425 Mbit/s) compared to the HEXITEC (1 Gbit/s) and has the advantage of larger frames reducing the frame-by-frame overhead in SpeXIDAQ, the CPU time used to convert raw frames to a photon event stream is not significantly lower compared to the HEXITEC. This is due to an additional processing step required to remove common mode noise typical of CCD sensors, which is fairly demanding.

## 7. Case Studies

In the following two case studies, SpeXIDAQ did not cause any bottlenecks, either in processing time or experimenter effort. In both cases the limiting factor in setting exposure times and the achieved amount of image statistics was the flux rate limitation of the used hyperspectral detector.

### 7.1. Case Study I: Beamline Diagnostics

Energy-resolved X-ray transmission imaging experiments are very commonly performed at synchrotron beamlines, where energy selection is usually not provided by the detector, but by having an extremely narrow bandwidth monochromatic X-ray source with tuneable energy. While these setups are very high performance in terms of monochromaticity and flux, no optics system is ever flawless. During a recent beamtime at Diamond Light Source I13-2, an L-edge subtraction experiment around the Au $L_{\mathrm{III}}$-edge ( 11.92 keV) was performed to image the distribution of gold nanoparticles inside biological samples. The expected signal was unfortunately dominated by the presence of vertical intensity fluctuations in the X-ray beam, which could not easily be compensated for.

These intensity fluctuations are a result of the multilayer monochromator (MLM) used for this experiment, and have been described before in literature [22,23]. These descriptions, as well as common normalization techniques, assume a perfectly monochromatic spectrum which is not guaranteed to be the case. In order to investigate whether these intensity variations are also accompanied by small fluctuations in beam energy, a second beamtime was requested where the beamline was characterized using the pnCCD-based SLcam camera and the SpeXIDAQ software system. While the full discussion of this experiment has recently been published [12], this work will highlight two key aspects where the SpeXIDAQ system played a key role in the experiment.

Over the entire beamtime a total of 43 measurements were taken, totalling 95 GiB of processed data and 3.4 GiB of raw detector output across 19 h of exposure time.

#### 7.1.1. Live View of Small Energy Fluctuations

Because the spatial energy variations in the beam—if any—are likely very small, an appropriate visualization method was required. This experiment is at the origin of the mean energy data output type implementation described in Section 4.1, which is very sensitive to minute energy differences, yet largely unaffected by intensity variations.

**Figure 8:**
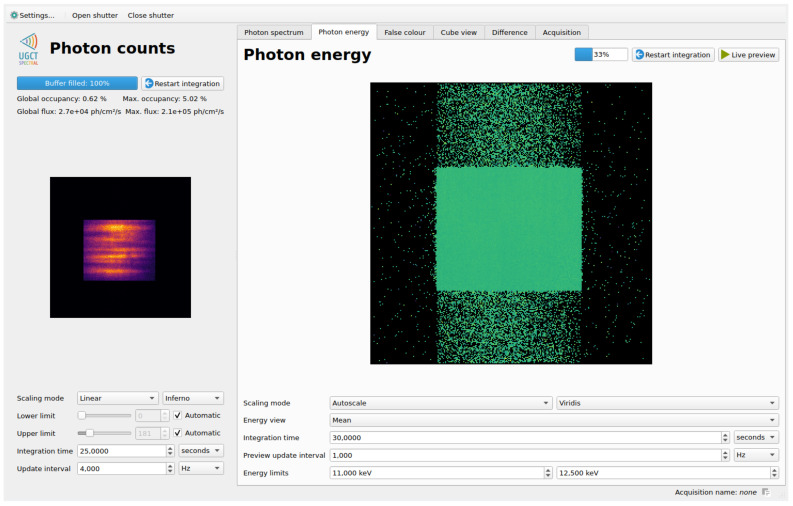
The main SpeXIDAQ GUI as used during a recent beamline diagnostics measurement. The integrated counts view on the left shows clear intensity variations caused by the monochromator, while the mean energy shows no fluctuations in the spectrum. The vertical distribution of signals above and below the main aperture is an artefact of the CCD readout. The flux in the illuminated region of the sensor was $2.1\times 10^{5}$ $\mathrm{ph}s^{-1}$ ${\mathrm{cm}}^{-2}$ for a pixel occupancy of 5%.

The live view presented in Figure 8 shows the integrated counts view on the left, and the mean energy in the 11 to 12.5 keV interval around the main spectral peak. The integrated counts view clearly shows the intensity variations inherent in the type of MLM used for this optics setup, while the mean energy view indicates a spatial energy distribution at least as uniform as the camera’s spectral sensitivity. Thanks to the live view which allows for decoupling energy variations from intensity variations, the team was able to optimize the limited measurement time of the beamtime towards getting the maximum amount of useful data for offline analysis.

#### 7.1.2. Camera Protection Using a Fast Shutter

The pnCCD-based SLcam is susceptible to radiation-induced aging, where detector performance will gradually decline over time. The high cost of the system requires the operator to keep incident flux at a minimum to prolong the useful life of the detector, and the incident photon power absorbed by the detector should stay within safe levels to prevent delamination of the cooling system or other physical damage to the sensor. These requirements mean that this type of camera system is not typically placed within the full X-ray beam of a synchrotron. Instead one uses extensive amounts of filtering, or experimental setups designed such that only a small fraction of the beam’s flux reaches the detector due to the geometry, optics, or the measurement technique itself.

To protect the SLcam while being placed in the full (filtered) beam of the I13-2 branchline at Diamond Light Source, a fast shutter was placed between the detector and the end of the beampipe. An extra process was added to the SpeXIDAQ pipeline, continuously monitoring the incoming photon flux on a frame-by-frame basis, and closing the shutter in case of an excess. Because of the relatively high framerate ( 400 Hz) of the SLcam and the quick mechanical operation of the shutter, any excessive flux could be cut off within milliseconds. This mechanism was triggered a number of times during the experiment, usually when an operator was changing the filter configuration and a set of filters appeared with too little attenuation.

The flux is calculated not by reconstructing charge clusters and setting a limit based on photon counts, but by counting the pixels over the noise thresholds in each frame, both globally, and with the frame subdivided in 64 smaller sections to catch local bright spots. In this experiment the limit was set at 10% pixel occupancy, with measurements generally aiming for 6% occupancy or lower.

This demonstrates the flexibility in SpeXIDAQ’s design, where in a short timeframe new utilities can be developed using intermediate data to handle experiment-specific tasks outside of normal operation.

### 7.2. Case Study II: Spectral Radiography Using a Multi-Sensor Camera

One of the supported hyperspectral X-ray cameras is the quad sensor tiled HEXITEC system. This camera uses four 80×80 pixel HEXITEC ASICs, with a roughly 500 μm gap between the tiles. To support multi-sensor cameras and other advanced systems, the frames used internally in SpeXIDAQ are virtual, and do not need to correspond to the physical frames exiting the hardware. This way the individual sensor outputs can be placed accurately within the overall frame, if desired even with rotations and shifts of less than one pixel. While in normal imaging operation these fractional pixel positions would get binned back to the regular pixel pitch, certain experiment-specific processing methods can benefit from a more precise approach, for example by placing the charge clusters on a grid with narrower pitch than the physical pixels when generating images from the reconstructed photon events.

**Figure 9:**
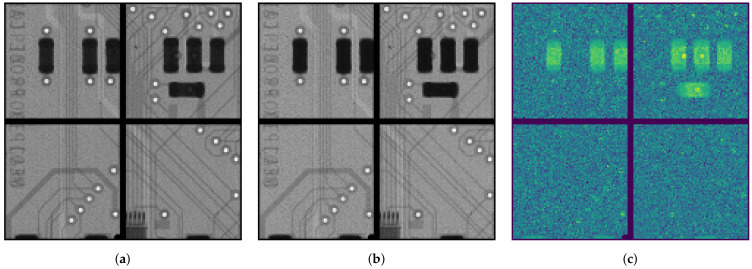
Spectral radiographs of a Medipix 2 board taken with a 2 × 2 tiled HEXITEC array and a white X-ray spectrum from a microfocus lab source set to a tube voltage of 50 kV. Integration time of the flatfield and sample images is 2 min, and the source flux was set to keep the flatfield pixel occupancy close to 6% for a flatfield flux of 2.8 × 10^5^ and 1.2 × 10^5^ phs^−1^ cm^−2^ through the sample. The CdTe HEXITEC was configured with a bias voltage of 300V, and a bias refresh of 5 s every 30 s. The peltier cooling was set to a 20 °C setpoint. (**a**) 1 keV interval below the Ba K-edge. (**b**) 1 keV interval above the Ba K-edge. (**c**) Difference image highlighting the location of barium.

To highlight the spectral contents of hyperspectral X-ray imagers and the use of tiled sensor arrays in the SpeXIDAQ system, a transmission view of a Medipix 2 board is shown in Figure 9. Two narrow energy ranges are shown, both 1 keV wide and placed above and below the 37.4 keV barium K-edge at center energies of 36.4 and 38.4 keV respectively. The difference of both images after normalization is shown in Figure 9c and clearly shows a signal in the ceramic capacitors, which often use barium as a dielectric in the form of a BaTiO_3_ compound.

While Figure 9 shows the offline processed data of a measurement taken using SpeXIDAQ, this presence of Ba is of sufficient concentration to also be visible in the live previews, showing their strength in on-the-fly material identification during an experiment. To generate these figures, two measurements were performed. One without sample to retrieve the flatfield illumination and spectrum, and one with sample. Both measurements used identical source, detector and data processing settings. In each case the data cube output type was used with 1000 bins between 0 and 100 keV. The measurements each required just under 100 of storage.

## 8. Discussion

The SpeXIDAQ software framework is a new data acquisition system for hyperspectral X-ray cameras. By design it combines three key aspects: a high performance backend ready for the next generation of hyperspectral X-ray sensors with ever increasing processing demands, flexible customization to fit the various measurement techniques and experiments that can benefit from energy information, and a powerful yet user friendly operation streamlined for actual scientific experiments. The resulting software has been refined over a variety of different experiments and beamtimes, validating its performance and stability. The inherent flexibility allows for quick adaptations to new measurement techniques, and integration into existing systems with minimal overhead and development time.

To sensor and camera developers the SpeXIDAQ system offers very extensive diagnostics and hardware debugging tools aiding in characterization, calibration, and the development of new hardware support plugins. This diagnostic output is also an advantage for end-users of these camera systems, which can use the extra information provided by the framework to fine-tune their experiments and get a better grasp of the specific challenges posed by the use of hyperspectral X-ray sensors. In many current offerings this wealth of information is missing, causing failed experiments or misleading data due to lack of understanding of the underlying hardware operation.

In closing, a remark should be made on adapting SpeXIDAQ for use with threshold-based multispectral PCD devices. As these sit somewhere between monochromatic PCDs and full-fledged hyperspectral systems, there could be some benefit to including them in the framework. This would simplify some of the processing steps (e.g., cluster reconstruction would no longer be required, nor possible). However, the data structures used for internal communication are optimised for use with hyperspectral data, and might not be the most efficient at carrying multispectral data through the system. For example, the photon event stream method of carrying the reconstructed events would be far less efficient compared to carrying the 2D frames of thresholded counts compared to the single events recorded by the direct measurements of hyperspectral sensors. The user interfaces would also need to be modified to represent the different data in a sensible manner, and to expose any functionality not required or provided by hyperspectral sensors, such as configurable energy thresholds.

## 9. Conclusions

Hyperspectral X-ray sensors require a different approach to data processing, visualization and overall system control. The SpeXIDAQ framework was developed to fulfill these specific needs, while remaining extensible and general enough to support different sensors and camera systems. Throughout multiple lab-based experiments and synchrotron beamtimes, the performance and applicability of SpeXIDAQ has been verified.

End-users benefit from increased camera performance, live feedback through graphical user interfaces, and a wealth of new processing options. Sensor and detector developers can take advantage of the existing framework to quickly include new sensors and camera systems, and have a toolbox of diagnostic utilities at their disposal for hardware characterisation and troubleshooting.

## Figures and Tables

**Table 1:** Hardware components in the workstation used to run all acquisition and on-line processing described in this work.

Component	Description
CPU	Intel i7-7700K @ 4.20 GHz, 4 cores 8 threads
GPU	Integrated Intel GPU, not used for data processing
RAM	32 GB DDR4 in four 8 GB modules, clocked at 2133 MT/s
Storage	Samsung SSD 750 EVO 250GB for operating systemVarying set of HDDs in raid configurations for data storage
Connectivity	HEXITEC: Intel 82571EB Gigabit Ethernet ControllerSLcam: National Instruments PCI-8336 PCI-to-PXI bridge (fiber optic version, supplied with camera)
Operating system	Debian 10.7 with 5.9.6 kernel

**Table 2:** Resource consumption by the SpeXIDAQ framework in active use, with a running measurement configured to include 4000 energy bins in the data cube output. The backend and GUI are listed separately, as the latter can be run on a different workstation, and has a high degree of variability in memory and CPU usage depending on viewing settings. All configuration and GUI settings are representative for a typical X-ray imaging experiment using a polychromatic lab source and optimised flux for low pile-up and acceptable statistics.

	Backend		GUI			
	CPU	RAM	CPU	RAM	Framerate	Flux
HEXITEC	25%	816 MiB	<1%	88 MiB	9 kHz	$3.7\times 10^{5}$ $\mathrm{ph}s^{-1}$ ${\mathrm{cm}}^{-2}$
SLcam	16%	2862 MiB	<1%	98 MiB	400 Hz	$1.8\times 10^{5}$ $\mathrm{ph}s^{-1}$ ${\mathrm{cm}}^{-2}$

## Data Availability

Readers interested in obtaining access to the SpeXIDAQ framework are encouraged to contact the authors. A collaborative effort to extend the application range and add new detector families to the set of supported hardware is open for discussion. While not the main focus of this work, the datasets used to generate the figures can be obtained through the corresponding author.

## Abbreviations

The following abbreviations are used in this manuscript:

MDPI	Multidisciplinary Digital Publishing Institute
DOAJ	Directory of open access journals
CCD	Charge Coupled Device
XRF	X-ray Fluorescence
CT	Computed Tomography
PCD	Photon counting detector
TTL	Transistor-transitor logic
GUI	Graphical user interface
CLI	Command line interface
RGB	Red, green and blue
MLM	Multilayer monochromator
ASIC	Application specific integrated circuit
